# The impact of nephrostomy balloon inflation volume on post percutaneous nephrolithotomy hemorrhage

**DOI:** 10.11604/pamj.2020.36.384.25162

**Published:** 2020-08-31

**Authors:** Firas Azar Khori, Mohannad Mueen Al-Naser, Ashraf Suleiman Al-Majali, Mohammad Abdelfattah Al-Serhan, Awad Bakheet Al-Kaabneh, Abdelhakim Saleh Ni’mate, Ayman Ahmad Al-Qaralleh, Abdullah Muhammad Alrababaah, Samer Gaith Al-Jfout, Nizar Jamal Al-Saidah, Ali Ahmad Al-Asmer, Belal Abdullah Al-Khawaldah, Monther Ata Alemoush, Anees Adel Al-Hjazeen

**Affiliations:** 1 The Prince Hussein Urology Institute (PHUI), The King Hussein Medical Centre, (KHMC), Amman, Jordan,; 2 Interventional Radiology (IR), The King Hussein Medical Centre, (KHMC), Amman, Jordan

**Keywords:** Percutaneous nephrolithotomy (PCNL), post-PCNL bleeding, nephrostomy balloon catheter

## Abstract

**Introduction:**

the study aims to match different volumes of nephrostomy balloon inflation to point out the foremost effective volume size of post percutaneous nephrolithotomy (PCNL) bleeding control.

**Methods:**

we have retrospectively reviewed “560” medical records of patients who underwent percutaneous nephrolithotomy between (the years 2017 and 2018) at Prince Hussein Urology Center. The Patients were divided into two teams, group-1 (a number of 280 patients) with nephrostomy balloon inflated concerning three ml and group-2 (a number of 280 patients) the balloon inflated concerning one ml. The preoperative and postoperative hematocrit, the operation duration, the stone size, the postoperative pain severity, the transfusion rate and the duration of hematuria between the two groups were compared during hospitalization.

**Results:**

regarding patients with ages (between 18 and 68 years); the preoperative hematocrit (mean values ± SDs) was (40.35% ± 3.57) vs (39.95% ± 3.43) for groups-1 and 2, respectively; the p value=0.066. The postoperative hematocrit was (37.91% ± 3.96) vs (34.38 ± 2.78), respectively; the p value was (0.008); the blood transfusion rate was 11.2% vs 13.4% (the p value was 0.039), respectively. The Postoperative pain score was (4.93 ± 1.44) vs (3.89 ± 1.45) (the p value was 0.012), respectively.

**Conclusion:**

increasing the nephrostomy balloon volume to a “3cc” competes for a task to decrease bleeding which was found to be as a secure and considerable effective procedure-related factor. However, the disadvantage of this technique resulted in increasing the postoperative pain in patients undergoing such a procedure.

## Introduction

Kidney stones area unit is a common malady that affects individuals from all the countries of the world; it has been found that over the past twenty years there has been a motivating increase in its incidence [1]. Renal calculi management has been dramatically evolved from open procedures to minimally invasive procedures. In 1941 Rupel and Brown succeeded in renal stone removal via Nephrostomy [2]; there have been nice enhancements in surgical techniques, surgical training and instruments. In 1976 Fernastom and Johansson performed the primary percutaneous nephrolithotomy (PCNL) [3]. PCNL is typically used for stone that is bigger than “20mm”, cases with stag-horn or cystine stones, hard stone that is not responding to ESWL or a stone which is associated with congenital kidney malformations [4]. Postoperative nephrostomy tube placement for drainage through the percutaneous tract has become a key step in PCNL; moreover, the nephrostomy tube enhances hemostasis on the tract and reduces urinary leak as well [5]. Despite that, PCNL is less invasive when compared to an open procedure; nevertheless, there are some complications; percutaneous nephrolithotomy is a successful, less invasive surgery (>90%) at the price of greater complications (>10%) [6]. There are some complications that may occur post PCNL, such as: bleeding, renal pelvis perforation, intraabdominal and intrathoracic organ injuries, sepsis, nephrocutaneous fistula, renal loss and death [7-10]. In this study, we are going to judge the degree of nephrostomy balloon inflation on post PCNL complications, principally bleeding.

## Methods

This retrospective study was carried out in the Royal Medical Services at the Prince Hussein Urology Centre; by reviewing the medical records of (560) patients who underwent PCNLs by multiple experienced urologists from the period between (January 2017 and December 2018), the patients had been found to suffer from renal stones sized between (1cm and 6.5cm) documented by imaging modalities like kidney, ureter and bladder plain X-ray (K.U.B), ultrasonography (US) and non-contrast computed tomography (renal CT stone protocol). Accordingly, the patients were classified into two equal groups in number of patients, group-1 (the number of patients was: 280) as well as patients with post PCNL nephrostomy balloon inflated concerning 3ml and group-2 (the number of patients was: 280) patients with nephrostomy balloon inflated concerning 1ml. In principle, patients who underwent PCNLs have had negative urine culture, stones that refractory to extracorporeal shock wave lithotripsy (ESWL), radiolucent stones, their ages were between 18 and 69 years; all were with normal coagulation profile and a creatinine level. Patients with uncontrolled hypertension, pregnant patients, with ureteric obstruction or suspicious renal mass, single or transplanted kidneys were excluded.

A prophylactic antibiotic (a third-generation cephalosporin ceftriaxone 1gm IV x 2) one day before the procedure and continued for 48 hours post the procedure, post the procedure was given to all patients. After a retrograde angio catheter six FR and Foley catheter 16 FR insertion with the patient in a lithotomy position and anesthetized; a tract was created by an interventional radiologist after the patient being in a prone position, using multidirectional C-arm fluoroscopic guidance. Alken´s coaxial dilators were used for tract dilatation to 30F. A rigid twenty-six F nephroscope (Karl Storz endoscope) was used through an Amplatz sheath. Ultrasonic or pneumatic lithotripters were used for stone disintegration, with forceps getting used for stone fragment removal. A Foley catheter sized 16 FR served as a nephrostomy tube was put at the end of the procedure and the balloon of this catheter was inflated with sterile water or normal saline (3cc for cluster one and 1cc for cluster two) and was kept for a period of 24-48 hrs. Patients were discharged on oral antibiotics (ciprofloxacin 500mg x 2 for 5 days). The patients were followed from the primary day post-procedure up to (2-6 months), with a mean follow-up period of four months for all patients. Patients underwent K.U.B, a non-contrast renal computed tomography scans and urine culture two weeks post any procedure, with a patient´s temperature being measured throughout admission. If the patient developed a fever, which was outlined by (temperature >37.5°c orally), urine culture was requested and the correct antibiotic was given with accordance to the culture results.

Both groups were compared regarding the demographic data, preoperative and postoperative hematocrit level, operation time, stone size and locations, postoperative fever, postoperative pain severity (pain score 1-10), blood transfusion rate and duration of the hematuria throughout hospitalization. Statistical analysis in reference to the previously mentioned variables between each team was done by using SPSS computer program version-24; the results were expressed as mean ± SD (standard deviation) or number, the comparison between the mean values of both groups continuous clinical variables was done by using Mann-Whitney U test. The comparison between both groups regarding the categorical data (N (%)) was done by the chi-square test. The P-value <0.05 was considered statistically significant. We attained approval from our institution in the Royal Medical Services ethical committee for publication.

## Results

In all, 560 patients were registered during the study. Of these patients, there have been 308 males (55%) and 252 (45%) females; stones were sized from (1 to 6.5cm) for all patients with a mean value ± SD: (3.023 ± 1.407cm, respectively); 177 (32%) patients had upper group calyxes´ stones; 148 (26%) middle group stones and 235 (42%) lower group stones. In Table 1; we have a tendency to place the demographic data of both groups regarding: age groups, genders, stone sizes and locations. The previous data have been presented as mean value ± SD and percentages with numbers in reference to the entire number of all patients. With reference to the categorical data of group one and group two, for example, preoperative and postoperative hematocrit value, operative time, postoperative fever and pain, blood transfusion rate and finally the duration of hematuria, we have conducted a comparison and placed the calculated mean value and standard deviation with the calculable P-value in Table 2. The preoperative hematocrit level for group-1 ranged from (33% to 50%) and as for group-2 from (36% to 47%); however, postoperative value was: from (27% to 49%) and from (28% to 42%) for groups-1 and 2, respectively. The operation time was: (30-90 minutes) and (32-89 minutes); the postoperative pain score was: (3-8) and (2-7) and the duration of hematuria was: (from 1 to 4 days) and (from 1 to 6 days); the blood transfusion rate was: (11.2% vs 13.4%), postoperative fever (7.4% vs 7.6%) for groups-1 and 2, respectively.

**Table 1:** demographic data of both groups

Variables	Group-1	Group-2
Ages(%)	18-63(50%)	20-68(50%)
Males(%)	157(28%)	151(27%)
Females(%)	123(22%)	129(23%)
Stone size (mean ± SD^®^)	1.3-6.5cm(3.1 ± 1.4cm)	1-6.3cm(2.9 ± 1.4cm)
Stone location: upper(%)	96(17%)	81(14.5%)
Middle(%)	72(12.9%)	76(13.6%)
Lower(%)	112(20%)	123(22%)

SD^®^: standard deviation

**Table 2:** the differences between both groups regarding the categorical data

Variables	Group-1	Group-2	The P value
Preoperative hematocrit level (mean value ± SD©)	(40.35% ± 3.57)	(39.95% ± 3.43)	0.066
Postoperative hematocrit level (mean value ± SD)	(37.91% ± 3.96)	(34.38 ± 2.78)	0.008
Operation time (mean value ± SD)\min	(68.11 ± 13.21)	(68.15 ± 12.88)	0.032
Postoperative fever (%)	(7.4%)	(7.6%)	0.006
Postoperative pain severity (mean value ± SD)	(4.93 ± 1.44)	(3.89 ± 1.45)	0.012
Blood transfusion rate (%)	(11.2%)	(13.4%)	0.039
Duration of hematuria (mean value ± SD)\days	(1.8 ± 0.75)	(3.7 ± 1.33)	0.023

SD©: standard deviation

## Discussion

As has been mentioned earlier in the results, the drop in hematocrit level was additional in group-1 (balloon inflated with “1cc” N\S), additionally; the blood transfusion rate was a lot of within the same cluster with longer hematuria period time, whereas, the postoperative pain was more in group-2 (balloon inflated with “3cc”). Once creating a comparison between each team in reference to the opposite variables, for example, operative time and postoperative fever, no important discrepancy between both groups was found; furthermore, no significant variations in stone sizes and locations. Raymond KO and associates reported that PCNL plays an integral role in managing most of the enormous renal stones with a wonderful stone clearance rate and least complication rate as well as the postoperative bleeding [11]. On the opposite hand, Foley catheter traction as a nephrostomy drain tube is effective and safe for reducing post-PCNL bleeding [12]. In an original article concerning the safest technique for management bleeding post-PCNL, Tahir Karadeniz *et al*. mentioned that the inflation of the nephrostomy balloon tube in the PCNL tract is an efficient technique for postoperative bleeding control [13]. Therefore, this supports our results regarding the post PCNL bleeding control by increasing the quantity of the nephrostomy balloon catheter inflation within the amount that not inflicting that a lot of important severe postoperative pain.

On the opposite facet, Wei-Hong Lai and colleagues reported that tubeless PCNL may be a safe modality in an exceedingly sensible hemostasis operation [14] which was additionally detected in an article concerning the security of passing over of nephrostomy drainage post-PCNL in the absence of clear indication [15]. Some urologists replaced the concept of increasing the inflated nephrostomy balloon size (tamponade effect) by covering the nephrostomy tube with absorbent hemostatic gauze (hemostatic effect) which was also an effective method in decreasing the postoperative bleeding [16]. Regarding the postoperative pain which was more in group-1 than in group- 2 due to the increasing volume of the inflated balloon in the former group; there was an identical study that compared the postoperative pain and complications among the variable sizes of nephrostomy tubes post PCNLs; this study documented that the larger sizes of nephrostomy tube increased the postoperative pain and complications over little size tubes and even tubeless procedures [17].

Conservative management of the delayed hematuria post PCNLs was the foremost effective choice for this rare and serious complication [18]. In our study, the foremost integral part of the conservative option for the treatment of prolonged hematuria period was increasing the degree of the inflated nephrostomy balloon catheter. The post PCNLs fever and sepsis weren´t a complication that is associated with the degree of the inflated nephrostomy balloon; as no significant differences were seen between both groups in our study. This complication relies on multiple risk factors in relation to the patients´ profile, stone's characteristics and anatomical, pathophysiologic and operative factors [19]. However, the other complications post PCNLs mainly the bleeding is expounded to multiple risk factors as were mentioned in several articles [20-23] and the size of nephrostomy inflated balloon.

## Conclusion

The post PCNL bleeding is dependent upon multiple risk factors which are associated with: the patient profile, stone characteristics and procedure factors. Whereas, the operative time and postoperative complications weren´t considerably littered with the degree of the inflated nephrostomy balloon tube, the controlling of postoperative hemorrhage was best achieved by increasing of the inflated balloon volume to a “3cc”. However, the disadvantage of this technique lies in the increase of the postoperative pain score which wants any analysis studies to work out the foremost effective inflated volume that its advantages outweigh its disadvantages.

### What is known about this topic

- Nephrostomy tube serves as a drain post PCNL;
- Nephrostomy tube can protect the kidney post the procedure from the ureteral obstruction;
- Nephrostomy balloon can compress the PCNL tract against bleeding.

### What this study adds

- The volume of the nephrostomy balloon affects the post PCNL bleeding;
- The increased volume of the nephrostomy balloon to a 3cc increase the post-operative pain;
- The operative time not affected by the balloon volume.
